# Companion animal organoid technology to advance veterinary regenerative medicine

**DOI:** 10.3389/fvets.2023.1032835

**Published:** 2023-03-17

**Authors:** Louis C. Penning, Robin van den Boom

**Affiliations:** Department of Clinical Sciences, Faculty of Veterinary Medicine, Utrecht University, Utrecht, Netherlands

**Keywords:** organoid, regenerative medicine, intestine, liver, disease modeling, companion animals, horse

## Abstract

First year medical and veterinary students are made very aware that drugs can have very different effects in various species or even in breeds of one specific species. On the other hand, the “One Medicine” concept implies that therapeutic and technical approaches are exchangeable between man and animals. These opposing views on the (dis)similarities between human and veterinary medicine are magnified in regenerative medicine. Regenerative medicine promises to stimulate the body's own regenerative capacity *via* activation of stem cells and/or the application of instructive biomaterials. Although the potential is enormous, so are the hurdles that need to be overcome before large scale clinical implementation is realistic. It is in the advancement of regenerative medicine that veterinary regenerative medicine can play an instrumental and crucial role. This review describes the discovery of (adult) stem cells in domesticated animals, mainly cats and dogs. The promise of cell-mediated regenerative veterinary medicine is compared to the actual achievements, and this will lead to a set of unanswered questions (controversies, research gaps, potential developments in relation to fundamental, pre-clinical, and clinical research). For veterinary regenerative medicine to have impact, either for human medicine and/or for domesticated animals, answering these questions is pivotal.

## Introduction

In ancient Egypt cats were considered to be sacred animals, demonstrated by the fact that mummified cats have been found near mummified people. When dogs became “man's best friend” is unclear but we know that the famous French philosopher Voltaire wrote “*c'est le meilleur ami que puisse avoir l'homme*,” which translates as “the best friend man can possibly have” in the eighteenth century. The history of interactions between cats and dogs and man has some defining elements. Since the first genetic evidence for an Eastern Asian origin was published in 2002 the origin and timing of canine domestication has been heavily debated, as a result of novel analytical and archeological findings (1–13). In addition to domestication, two more recent factors have been the driving force behind feline and canine genetic research and high-quality medical care for pets. The first was the selective inbreeding of these animals, especially dogs, that started in the middle of the nineteenth century, seeking to optimize specific physical and behavioral traits needed to perform as working animals, as was common in those times (14). A second stimulus was the change in the appreciation of pets and horses, they became members of the family. Combined with a much-improved financial situation after the end of the Second World War, this led to an enormous boost in initiatives to bring veterinary medical care of pets to a very high level. Consequently, veterinary (medical) knowledge and technology available for diagnostics and treatment specifically for pets, has become very comparable to that available for man. As a result, the “One Medicine” concept arose which implies that therapeutic and technical approaches are exchangeable between man and animals and can be used for their mutual benefit. Here we focus on adult stem cell-biology and organoid technology in veterinary Regenerative Medicine (RM) within the “One Medicine” concept. This review focuses on adult, organ-specific stem cells and the organoid cultures derived from them and the implications for disease modeling and potentially cell/organ replacement. For a recent review of farm and domestic animal organoids in food safety and public health (zoonotic diseases) readers are referred elsewhere (15).

## Research strategy

The latest PubMed search was performed on Monday September 19th. Searching for organoid^*^ AND (canine OR equine OR feline OR horse^*^ OR dogs OR cats) revealed 457 hits (see Table 1). Inclusion of a time period from January 1st 2005, years before LGR5 was established as a hallmark of epithelial stem cells (16) and the creation of organoids from single LGR5+-cells (17) reduced the number to 91 hits and as such this Pubmed search revealed only organoids as defined by Lancaster and Knoblich (18). After individual analysis of these 91 papers, some were discarded for several reasons, most often the fact that the research had been conducted using the Madine Darby Canine Kidney cell line. This selection resulted in fewer than 50 papers, all of which are included in this review. To visualize the negative selection in this last round, the main reason for exclusion from this review is presented in Supplementary Table 1. A similar research strategy with (regenerative medicine) AND (canine OR equine OR feline OR horse^*^ OR dogs OR cats) and further selection based on abstracts and content produced a very similar list of publications.

**Table 1:** Pubmed search strategy (latest search Monday September 19th, 2022) selection.

Organoid* AND (canine OR equine OR feline OR horse* OR dogs OR cats)	457 hits
# plus from January 1rts 2005 onwards	91 hits
Individual selection (see Supplementary Table 1)	38 hits

Furthermore, we restricted our selection to LGR5+ or Wnt-signaling dependent adult stem cell derived organoids because of the crucial role of LGR5 and Wnt in the culture of adult epithelial stem cells (19).

## Is stem cell biology in veterinary medicine different from that in human medicine?

There does not appear to be an undisputed definition of “regenerative medicine” (RM) but, at the very least, it includes a process in which damaged or lost specialized tissue is artificially replaced by the proliferation of undamaged specialized cells. This definition is species independent and the inclusion of the word artificially discriminates RM from naturally occurring processes such as axial regeneration in amphibians, or antler regrowth in ungulates. In cell-mediated regenerative medicine stem cells, either pluripotent stem cells or adult stem cells (ASCs), are the workhorses of regeneration.

Adult (or organ specific) stem cells have a limited differentiation potential, restricted to (some) cell types of the organ in which they reside. Their activity is mediated by the so-called stem cell niche. The stem cell niche is a functional and organizational structure that provides signaling cues for cellular maintenance in a quiescent or toward an activation status. These stem cells are responsible for the replacement of cells during physiological cellular homeostasis, such as the rapid renewal of the intestinal lining (20). In addition, these cells become activated in case of severely hampered replacement of damaged cells, as is the case in chronic liver failure (21).

*In vitro* experiments require cells that resemble the physiology that one is interested in. One of the drawbacks of established cell lines is that these are often tumor-derived, and as such do not directly reflect physiological processes. On top of that, their oncogenic potential hampers applications in regenerative medicine. ASCs can overcome these limitations because ASCs can be harvested and cultured form healthy and diseased tissues. The culture of ASCs has skyrocketed since organoid cultures were developed (22). By definition, organoids are “*a collection of organ-specific cell types that develops from stem cells or organ progenitors and self-organizes through cell sorting and spatially restricted lineage commitment in a manner similar to in vivo*” (18). Three aspects of this definition deserve attention: (i) the cells of origin can be either pluripotent or ASC, (ii) not all cell types of the represented/donor organ need to be present or arranged as in the original architectural pattern within an organoid, (iii) no functional similarities with the represented/donor organ are mentioned. Organoid technology received a boost following the landmark discovery and subsequent identification of small intestine and colon stem cells which express the marker gene product LGR5 (16). Two years later single sorted LGR+ stem cells were shown to self- organize into crypt-villus like intestinal organoids (17). The transmembrane protein LGR5, involved in Wnt-signaling, turned out to be a classic adult stem cell marker for epithelial cells (18, 20).

Given that the regulatory signaling pathways in stem cell biology are highly evolutionary conserved it is expected that the creation of organoids from non-human or rodent species would be an easy task. However, the number of papers describing companion animal derived organoids is limited compared to mouse and human organoids [cfr ref. (22) with Table 1]. Tools to study species specific ASCs might be the culprit. The lack of convenient and validated tools to detect LGR5 expression in canine and feline tissues hampered the rapid extrapolation of data from man and mouse to veterinary biomedicine. Immunohistochemical staining of canine hair follicles showed immunopositive cells, but the specificity of the antibody was not confirmed with recombinant proteins (23). Rabbit polyclonal antibodies against LGR5 were used in a study on canine epithelial skin tumors (24), the overt cytoplasmic staining is in line with staining with validated antibodies in human cancers. This however is not sufficient selective to sort individual cells based on plasmamembraneous LGR5-expression. Therefore, experiments with single cell sorted LGR5+ human and rodent ASCs cannot be repeated in pets and horses. This drawback forced researchers in veterinary medicine to use culture conditions in which the ASCs were specifically selected based on the presence or absence of (often human) growth factors. In order to advance veterinary regenerative medicine and its crucial role in the “One-Medicine” concept, knowledge, and appreciation of the (inter-)species differences are of utmost importance.

## Organoids implemented in disease modeling and transplantation/replacement studies

**Figure 1:**
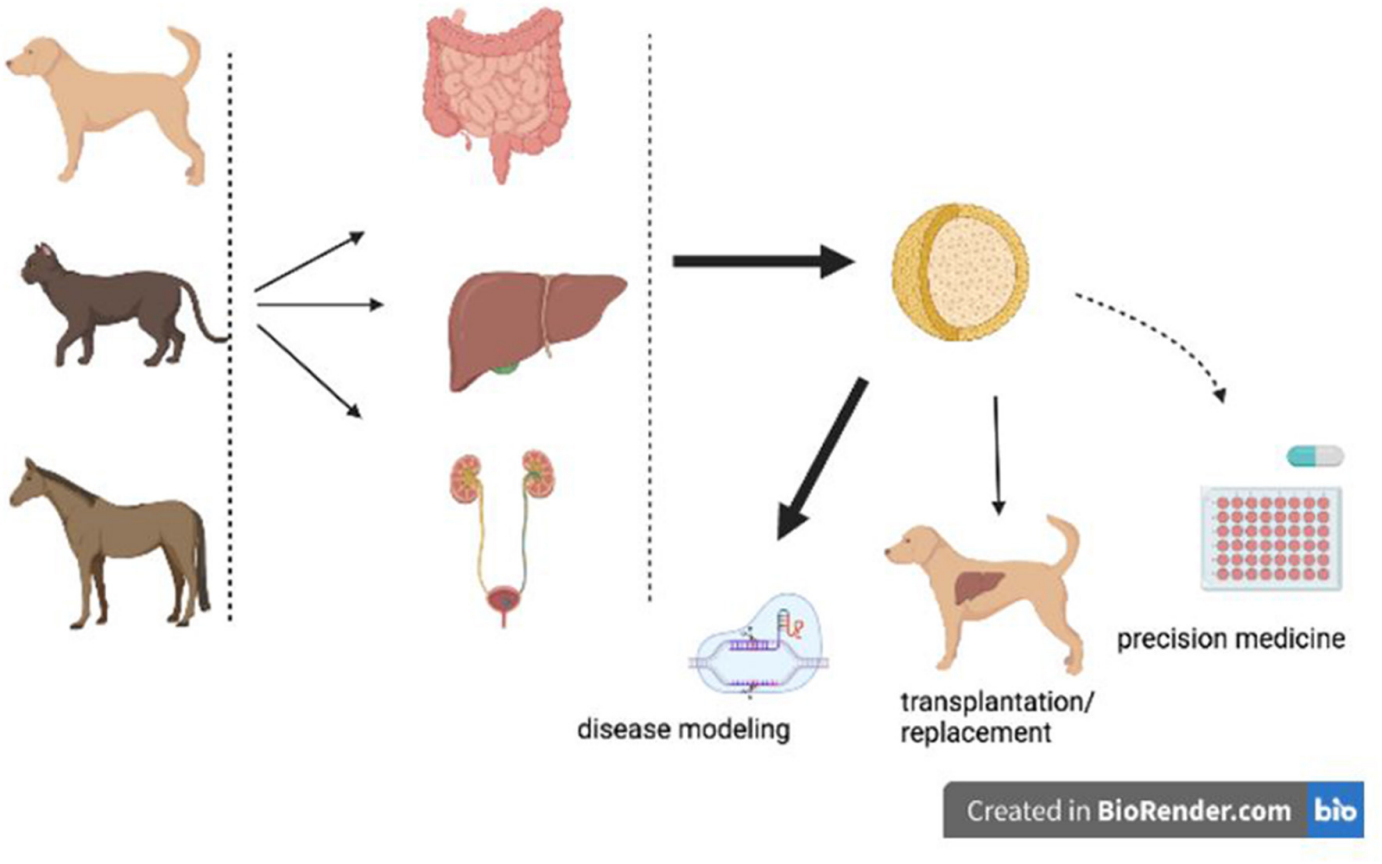
Potential and current applications of organoids in veterinary medicine. Organoids derived from various canine, feline, or equine organs can be used for disease modeling, precision medicine, or organ transplantations/replacement. Disease modeling is performed (fat black arrow), transplantations based on organoid technology is in a pre- clinical phase (thin black arrow), whereas precision medicine is on the horizon (dashed arrow).

Adult stem cells reside in an organ and can give rise to several of the cell types of that organ. The potential applications for adult stem cells in veterinary medicine include disease modeling and transplantation (Figure 1). These two applications come with their own specific points-of-concern, which can be partially addressed by means of organoid technology.

### Disease modeling

The importance of organoids in disease modeling is based on the fact that disease specific organoids can be created from biopsies (or fresh cadavers) from individual patients.

Moreover, a potential disease-causing mutation can be corrected, allowing cause-effect studies. The artificial culture conditions, with excess growth factor(s), oxygen and other nutrients, and the lack of interaction with other cell types (stem cell niche), means that some caution should be exercised when *in vitro* results are compared to *in vivo* data.

### Transplantation/replacement

Organ donation in veterinary medicine comes with ethical, logistical, and financial challenges. More realistic seems the application of organoid technology in veterinary medicine to provide predictive large animal models for human transplantations, given the similar size, environmental exposure, and life expectancy of pets and man, which are far more similar than rodents and man. This technology can be applied in a cross-species, intra-species or even autologous fashion (25, 26).

## Feline and canine organoids derived from adult stem cells

### Intestinal organoids

In 2009 a study was presented in which an ileal derived organoid transplantation was performed in six Beagle dogs (25). Little information was provided on the cellular composition of the transplanted organoids, and autologous transplantation did not lead to any neomucosa formation, irrespective of the location of the implant, in omentum or debrided intestine. In contrast, allogeneic organoids derived from fetal intestine resulted in mucosa formation only at the omentum implantation site. This positive result was found in three out of four dogs. It seems that no further investigations along these lines of research have been reported subsequently, although the search for canine intestinal organoids has continued.

A more detailed description of the establishment of intestinal organoids from domesticated animals was presented in 2017 (27). The long-term culture of intestinal organoids from various mammals, including cats, dogs, cows, horses, pigs and sheep was shown to depend on Wnt3a, R-spondin, and Noggin (27). This important observation confirmed the conservation of the signaling molecules needed for expansion of intestinal organoids. Quantitative RT-PCR results indicated *LGR5* mRNA expression, which confirms the importance of Wnt and R-spondin in the culture media. Unfortunately, the authors did not elaborate on the cellular differentiation of the stem cells toward other crypt and villus cell types.

Differentiation and more functional studies on canine intestinal organoids were centered along inflammatory bowel disease and intestinal (28–34). In line with the group's interest in understanding gastrointestinal diseases, intestinal organoids were cultured from healthy dogs, and dogs suffering from inflammatory bowel disease and intestinal adenocarcinomas. Crypt-villus structured organoids were derived from duodenal, jejunal, ileal, and colon regions, expressing markers for stem cells (*LGR5* mRNA in the bottom of the crypt), enteroendocrine cells, tuft cells and Paneth cells. Furthermore, the authors demonstrated the presence of a functional cystic fibrosis transmembrane conductance regulator (CFTR) and uptake of the parasite *Ascaris suum* derived vesicles (30). Inflammatory bowel disease was modeled in these canine colon organoids by means of LPS stimulation, which resulted in the differential expression of several tumor-associated genes, suggesting a mechanistic link between chronic inflammation and carcinogenesis (31).

Organoids derived from intestinal biopsies were used to investigate the differences in tight junction protein expression between dogs with protein losing enteropathy and healthy dogs (32). *In situ* hybridization (ISH), a way to measure mRNA expression in tissue slides, clearly showed enhanced *zonulin-1* mRNA levels in affected dogs compared to healthy dogs. As zonulin-1 negatively affects the cell-cell junctions, this observation explains the enhanced permeability (leakage) of the epithelial barrier which results in protein losing. From a stem cell marker perspective, this paper showed strongly elevated *LGR5* mRNA levels in enteropathic organoids. RNA-ISH circumvented the practical problem associated with the lack of validated anti LGR5-antibodies, by measuring mRNA levels rather than protein expression.

Together these in-depth molecular and functional descriptions of canine intestinal organoids pinpointed the important applications of organoid technology in disease modeling. The intestinal organoids form crypt-villus structures with the intestinal lumen on the inside. As such, this biologically relevant architecture hampers studies on transepithelial transport. To facilitate such studies and to measure microbial interactions the colon-derived organoids were cultured on a porous membrane in a polarized 2D-fashion (33). Cellular differentiation occurred, tight- and glycocalyx- junctions were formed, and the polarized cells expressed functional P-glycoprotein at the apical membrane. This adapted organoid system is deemed very well-suited for fundamental transport studies and disease modeling. A detailed protocol was presented on how to use canine organoids in a dual-chamber system permitting other researchers to create a similar experimental set-up for their own disease or species of interest (34).

Around the same time an international collaboration established and characterized long- term canine intestinal organoids derived from duodenum, jejunum, and colon (35). Although detailed gene expression analysis on cellular differentiation was described, and novel differentiation media compositions were provided, functional studies as presented by Allenspach's group were missing. This showed that small adaptations in the composition of organoid culture media need to be carefully described and species differences indicated.

Finally, one publication described feline intestinal organoids from ileum and colon aiming to study viral infections (36). Feline coronavirus infected only the colon-derived organoids, while the ileum-derived organoids remained uninfected.

All of the above studies investigated one or more aspects of the intestine using organoid technology. Little information is available to provide a more global analysis and comparison of gene expression levels and functional studies in relation to the most often used Caco-2 cell line (37). Both ATAC-Seq (open chromatin sequences) and RNA- Seq (expression analysis) was performed in human intestinal organoids and compared to Caco-2 cells. While the organoids' gene expression was enriched for transport systems and responses to oxidative stress, the Caco-2 cells highly expressed genes related to extracellular matrices. Interestingly, intestinal organoids reflected transport activity similar to the tissue of origin, even better then Caco-2 cells (38). Such in-depth molecular and functional studies are lacking in veterinary medicine, but the data from human intestinal organoids are promising. It remains to be seen how veterinary intestinal organoids can replace animals in pharmacological and toxicological studies related to intestinal tissue.

In summary (Table 2), canine and feline intestinal organoids can be created from most regions of the GI-tract and adaptation of the 3D-culture to a 2D system (cell polarization on a porous membrane) makes these cells ideally suited for disease modeling, especially because organoids can be created form biopsy material derived from animals with proven intestinal disorders.

**Table 2:** Summary of publications on intestinal organoids from dogs, cats, or horses.

**References**	**Species**	**Marker**	**Aim/disease**	**Remarks**	**Proliferation**
Agopian et al. (25)	Dog	N/A	Transplantation	Neomucosal formation	N/A
Powell and Behnke (27)	Numerous	LGR5	Disease modeling	Proliferation not differentiation	Wnt3a, R-Spo-3, and Noggin
Mochel et al. (28)	Numerous	N/A	Are pets preclinical models?	Commentary paper	N/A
Kopper et al. (29)	Numerous	N/A	Model for IBD?	Review paper	N/A
Chandra et al. (30)	Dog	a.o. LGR5	Pre-clinical model	Functional swelling assay	Wnt3a, R-Spo-1, and Noggin
Sahoo et al. (31)	Dog	PROM1 and OLFM4	IBD model	Gene expression after LPS stimulus	Wnt3a, R-Spo-1, and Noggin
Allenspach and Iennarella-Servantez (32)	Dog	LGR5 and ZO-1	Protein-losing enteropathy	Overview	N/A
Ambrosini et al. (33)	Dog	Low LGR5 and high NEUROH3	Generating monolayer of organoids	Detailed description of polarized cells	Wnt3a, R-Spo-1, and Noggin
Gabriel et al. (34)	Dog	N/A	Generating monolayer of organoids	Methodology paper	Wnt3a, R-Spo-1, and Noggin
Kramer et al. (35)	Dog	a.o. LGR5, PROM1, and NEUROG3	Disease modeling	Proliferation and differentiation	Wnt3a, R-Spo-1, and Noggin
Tekes et al. (36)	Cat	Low LGR5 and high MUC2	Disease modeling (viral infections)	Viral infection possible	Wnt3a, R-Spo-1, and Noggin
Stewart et al. (39)	Horse	a.o. LGR5	Disease modeling	Proliferation and differentiation	Wnt3a, R-Spo-1, and Noggin
Hellman (40)	Horse	a.o. SOX9 and MUC2	Disease modeling	2D organoids	Wnt3a, R-Spo-1, and Noggin

N/A, not applicable, not described; IBD, Inflammatory Bowel Disease; LPS, LipoPolySaccharide.

Human recombinant growth factors (Wnt3a, R-Spo-1/3, and Noggin) are used.

### Liver organoids

The first description of canine liver organoids was published in 2015 when hepatic stem cell derived organoids from COMMD1-deficient dogs were characterized (41). This paper reported the development of canine (liver) organoids and the authors presented evidence that the genetic defect leading to COMMD1-deficiency and subsequent hepatic copper accumulation was functionally restored by lentiviral transduction of the full coding sequence of the *COMMD1*-gene. These gene-corrected autologous hepatic stem cells were subsequently transplanted into recipient COMMD1-deficient dogs through the portal vein and survived for at least 2 years, but following engraftment in the liver hardly any proliferation of these cells was observed and no functional recovery with regard to copper accumulation and biliary copper excretion was observed (26). Of interest was the observation that organoids derived from healthy livers and liver from dogs with congenital portosystemic shunts (CPSS) were able to accumulate lipids if grown under conditions of excess free fatty acids in the medium, and as such resembled the steatosis as often observed in CPSS livers (42). As another example of disease modeling, canine liver organoids from healthy and COMMD1-defcient dogs and repaired COMMD1-deficient organoids were shown to have reduced FXR transcriptional activity, which is in line with observations in copper laden human livers (43–45).

Since cats, as obligate carnivores, may develop a peculiar hepatic lipid accumulation under stress (e.g., anorexia) and lipid-overload conditions, feline liver organoids were created and characterized in relation to feline lipidosis (46). Intra-cellular lipid droplets, both small and large, were formed if the organoids were cultured in high fat medium (0.4 mM oleic acid and 0.2 mM palmitic acid). This led to the establishment of rapidly growing feline liver organoids as an *in vitro* model for feline lipidosis, which was exploited to investigate the potential of several drugs to decrease hepatic lipid concentrations (47). Two candidate lipid lowering drugs were identified: T863 (a DGAT1-inhibitor that inhibits TAG synthesis) and AICAR, which seemed to mediate its lipid lowering effect by a decrease in *PLIN2* mRNA, a gene product responsible for lipid droplet formation [reviews on lipid droplet formation see Fader Kaiser et al. and Scorletti and Carr (48, 49)].

Given the gene expression and functions of some aspects of liver organoids, this system holds great promise for advancements in veterinary pharmacology as contributors to 3R (replace, reduce, and refine) policies. However, a cautionary mote was recently published on the hepatocytic phenotype of liver organoids (50). A comparison of numerous publications on transcriptome analysis and functional studies revealed that the hepatocyte-like cells in liver organoids derived from intrahepatic bile ducts or pluripotent stem cells (PSC) did not match the transcriptome of primary hepatocytes (gold standard). The intrahepatic bile duct or PSC-derived organoids more closely resembles cholangiocytes and HepG2 cells were actually more similar to primary hepatocytes. This would mean that the biotransformation potential of liver organoids might not be predictive for these activities *in vivo*. Although this study compared data from over 20 publications, canine, feline or equine liver organoids were not included. It is however conceivable that for these latter organoids the transcriptome and hepatocyte functions will also differ from those of primary hepatocytes, at best mimicking the *in vivo* situation.

Together (Table 3), the work on canine and feline liver organoids has proven these organoids' potential as disease modeling systems and has provided important lessons for large animal models of autologous stem cell transplantations.

**Table 3:** Summary of publications on hepatic organoids from dogs or cats.

**References**	**Species**	**Marker**	**Aim/disease**	**Remarks**	**Proliferation**
Nantasanti et al. (41)	Dog	a.o. LGR5, PROM1, and SOX9	Copper storage disease modeling	Long term expansion, and bipotential differentiation	Wnt3a, R-Spo-1, and Noggin
Van den Bossche et al. (42)	Dog	a.o. LGR5, PROM1, and SOX9	Congenital portosystemic shunt modeling	Lipid profyling and intra-organoid lipid accumulation	Wnt3a, R-Spo-3, and Noggin
Wu et al. (43)	Dog	a.o. LGR5, PROM1, and SOX9	Copper storage disease modeling	COMMD1 -/- organoid and reconstituted functional COMMD1 organoids	Wnt3a, R-Spo-1, and Noggin
Kruitwagen et al. (46)	Cat	a.o. LGR5, PROM1, and BMI1	Disease modeling: lipidosis	Long term expansion, and bipotential differentiation	R-Spo-1 and Noggin
Haaker et al. (47)	Cat	LGR5	Drug screens for lipidosis	Lipid droplet formation cane be reduced by specific drugs	R-Spo-1 and Noggin
Kruitwagen et al. (26)	Dog	a.o. LGR5, PROM1, and SOX9	Copper storage disease stem cell transplantation	Autologous stem cell transplantation with COMMD1 -/- organoids where functional COMMD1 was reconstituted	Wnt3a, R-Spo-1, and Noggin

N/A, not applicable, not described. Human recombinant growth factors (Wnt3a, R-Spo-1/3, and Noggin) are used.

### Skin organoids

The skin is probably the largest organ of the body, providing a barrier against external stressors and at the same time responsible for drug uptake. Moreover, scar tissue formation in the skin due to injury has social impact and affects quality of life (51). This has driven skin organoid research and the establishment of canine skin organoids (52, 53). Twelve dogs provided samples to establish and characterize canine skin organoids (51).

Immunohistochemistry and gene expression profiling confirmed that stem cell-based organoids were proliferative in expansion medium (Wnt stimulation and BMP inhibition) and in differentiation medium (i.e., neither R-spondin nor Noggin), with the typical cell types of the layered skin being expressed, as evidenced by a shift in keratin family member expression. An interesting observation was the differential mRNA expression of *lgr5* and its relative *lgr6*, viz lgr5 mRNA was only detected in hair follicle tissue and not in organoids, whereas *lgr6* was expressed both in hair follicles (HF) and intrafollicular epidermis (IFE) organoids at early passage. This is indicative of the different stem cell pools in HF isthmus, IFE and sebaceous glands in rodents (54).

To further optimize canine skin organoids representing the different epidermal cell layers culture media were adjusted (53). The most surprising finding was that IFE-derived organoids cultured in expansion medium (EM) resembled the layered skin architecture better than those cultured in differentiation medium (DM; addition of Wnt3a but not R- spondin or Noggin). Maybe the lack of Wnt proteins in the EM triggered some differentiation but further proof is needed to confirm this.

These EM-skin organoids are therefore suitable for disease modeling (e.g., atopic dermatitis) but when cultured on a porous membrane can be also used to study transepidermal drug uptake (33).

### Neuronal organoids

Brain biopsies are not performed routinely, for obvious reasons. Therefore, most brain organoids are based on induced Pluripotent Stem Cells (iPSCs) differentiated into mini brains (55, 56). *Post-mortem* material from five dogs was dissected to harvest canine hippocampal neural precursors (57). The organoids, or more precisely neurospheres, as the cells grow as condensed 2D cultures, proliferated and upon differentiation expressed neuronal markers (beta-tubulin) and glial cell (glial fibrillary acid protein, GFAP) markers. Differentiation was dependent on the addition of brain derived growth factor (BNDF). Fetal spinal cords were dissected from canine embryos at 40 days of gestation and the neurospheres differentiated into GFAP+ and tubulin+ cells, indicative of neuronal stem and progenitor cell maturation (58). These models are important given that dogs can suffer spinal cord trauma and develop a plethora of neurological disorders, including epilepsy and narcolepsy (59, 60).

### Cardiac organoids

The use of canine and feline models to stimulate “innovations” in cardiovascular research was recently strongly advocated (61) and extends beyond Doberman dogs with dilated cardiomyopathy or cats with cardiomyopathy (62, 63). Canine cardiosphere-derived cells (CDCs), cultured as 3-D organoids, differentiated into cardiomyocytes, smooth muscle cells, and endothelial cells *in vitro* (64). Moreover, canine CDCs injected intravenously in mice with doxorubicin-induced dilated cardiomyopathy engrafted in the heart, reduced fibrous tissue formation, and improved the cardiac capillary network.

### Renal organoids

The most frequently used canine cell is probably the MDCK (Madine-Darby Canine Kidney) cell line. In contrast to this famous cell line, important in the study of cellular polarization, only one publication exists on canine kidney organoids (65). Multipotent cells from one canine kidney have Mesenchymal Stem Cell (MSC)-like properties (adipogenic, chondrogenic, and osteoblastic differentiation) and, once grown in Matrigel, formed tubule-like structures. Further differentiation toward several renal cell types was not reported and the MSC-like phenotype points more to an MSC than an adult kidney stem cell, although CD24 and CD133 expression is in line with characterized renal ASCs. Renal ASCs express, amongst others, CD24 and CD133 when cultured in medium with Wnt-stimulators but have no adipogenic, chondrogenic or osteoblastic differentiation potential (66, 67). This means that canine or feline renal SCS have not yet been unequivocally established.

### Endometrial organoids

Lastly, feline endometrial organoids were polarized and expressed laminin in the basement membrane (68). Unfortunately, as this organoid was developed to assess plastic toxicity, little more information was provided on cellular differentiation or as a potential source of novel antibiotics, given the exposure of this organ to external microorganisms.

### Prostate cancer organoids

Like humans, dogs can develop prostate cancer and in both species this disease carries a poor prognosis. In view of the One Medicine concept spontaneously formed canine prostate organoids can be used as a translational model. The surprising source of the cells used to develop prostate cancer organoids was urine of 8 middle-aged to old dogs with various TNM classifications, which can be obtained non-invasively (69). Grown in Matrigel with medium composition as in man (including R-spondin and Noggin) the cells formed 3D luminal structures. The organoids expressed the epithelial marker E-cadherin, the myofibroblast marker alpha-SMA, basal cell marker CK5 and luminal cell marker CK8. When injected in immunodeficient mice these organoids formed tumors. All this indicated that the urine- derived prostate cancer cells behave like prostate cancer cells *in vivo*. Lastly, these cells were subjected to various chemotherapeutics in order to validate this system for disease modeling.

### Mammary cancer organoids

Canine mammary organoids can be derived from mastectomy samples cultured in Matrigel in FBS-supplemented DMEM-Glutamax/Hams F12 medium (70). The expression of CK18 (epithelial and adenocarcinoma marker) and CK14 (myoepithelium) confirmed the cancerous phenotype of these tumor organoids. No further studies on chemotherapeutic sensitivity and tumor formation in nude mice have been reported. As neither FBS nor the defined organoid media with Wnt, R-spondin, and Noggin was used, a comparison with other organoid systems cannot be made.

### Bladder cancer organoids

Four papers describe the establishment of canine bladder tumor organoids and the implications as models for drug response prediction (71–74). Typical epithelial and urogenital markers, such as E-cadherin, CK7 and CK20, were expressed. Moreover, the expression of vimentin and alpha-SMA indicated a more mesenchymal phenotype. Functional studies including the formation of tumors of injected organoids into NOD/SCID immunodeficient NOD/SCID mice, confirmed the cancerous phenotype of the bladder tumoroids. Finally, sensitivity to chemotherapeutics, specifically trametinib, was evaluated. All these data provided credence to the fact that these canine bladder tumor organoids resemble bladder tumors *in vivo* and might serve as *in vitro* models to evaluate drug responses on an individual level. As for the mammary tumors FBS was used as source of growth factors.

### Thyroid cancer organoids

Follicular cell thyroid organoids were created form healthy and primary thyroid gland tumors (75). As for most 3D organoid cultures Noggin and R-spondin were needed for propagation of the organoids in Basement Membrane Extract (similar to Matrigel and also animal- derived). The expression of TSHR (TSH receptor), NIS (sodium iodide symporter), and TPO (thyroid peroxidase) was similar in the organoids and in the primary tumors. No other functional studies have been reported.

For canine bladder and prostate cancer, the organoid models seem to be representative of the tumor *in vivo*. Thyroid cancer organoids and mammary tumor organoids require more functional studies and comparisons with the behavior of the original tumor before these tumor organoids can be considered as One Medicine models. Although counterintuitive, culturing 3D organoids from tumor material is sometimes more complicated than culture from healthy adult stem cells. First, the advantage of growth factor independent growth of tumor cells is lost in the rich (yet defined) culture media of 3D organoid cultures. In addition, the 3D structure inside the Matrigel, which is expensive and prone to batch-to-batch variations, requires long pre-culture processing and experienced handling. To overcome some of these limitations a so-called 2.5D organoid culture system was recently developed in which 3D organoids are cultured on top of (rather than as separate organoids) within a thick Matrigel layer (74). A further improvement in the tumor organoid field was the creation of these 2.5D structures without the need of 3D pre-culture (76). Applying this system, it seems possible to create tumor organoids and to study veterinary cancer biology, yet it is still dependent on Matrigel. A comparison with 2.5D organoids and similar tumors in DMEM showed enhanced proliferation in the organoid culture system. It remains to be seen how 2.5D on top of Matrigel organoids compare to 3D inside Matrigel organoids with regard to genetic stability (especially important for tumor-derived tissue), drug-sensitivity. For disease modeling this comparison must include differentiation potential to the required organ specific cell types.

### Corneal organoids

Corneal blindness affects both man and dogs. Initial work on corneal epithelial cells established the expression of progenitor marker p63 (77). Cultured on murine 3T3 fibroblasts as feeder cells, the canine corneal epithelial cells proliferated but did not form tumors (78). True corneal organoids have recentlty been described, although only one dog and one cat were used as donors (79). Here, p63 expression was confirmed only in the canine organoid, but LGR5 expression was no detectable, neither in the dog or the cat. The low number of donors and the lack of LGR5 expression clearly indicate that this organoid system needs further evaluation with regard to stemness and differentiation potential of the corneal stem cells.

## Equine adult stem cell derived organoids

Horses can be considered to be companion animals in many respects and are often treated by equine veterinary specialists. In large parts of the world horses, and other equids, are still used as working animals, whereas in the Western world *Equus caballus* is mainly used for sport and leisure. Horses are highly relevant large animal models for musculoskeletal disorders (osteoarthritis, tendinopathies) (80). Horses are frequently referred to veterinary clinics with gastro-intestinal disturbances, in particular colic, which refers to any cause of pain emanating from the gastro-intestinal tract (81, 82). Given the fact that *in vivo* studies in horses pose many logistical challenges related to the size (and cost) of the animals, but also related to standardization of conditions, one would anticipate an enormous commitment to establish equine organoids of various organ systems for *in vitro* research. Whatever the reason, only three papers describing the establishment and characterization of equine intestinal organoids have been published (27, 39, 40). Growth of these organoids, as in all intestinal organoid cultures, is Wnt dependent and upon differentiation various villus cell types, such as Goblet and Paneth cells, are formed. Important is the observation that these organoids can be cultured in a 2-D Transwell system permitting the study of microbial interactions with the luminal side of the epithelium. In 3-D organoid cultures the intestinal lumen is on the inside, which poses logistical hurdles when trying to expose these cells to microbials (82). Two papers describe equine oviductal and endometrial organoids to facilitate investigation of reproduction pathologies (83, 84). The creation of endometrial organoids from a Przewalski horse actually represents the first organoid of an endangered species (84, 85). This important observation proves the possibilities (and therefore novel opportunities) to investigate biological process in endangered species without the need to sacrifice these animals. Equine mammary organoids were functionally characterized and, importantly, prolactin induced the production of beta-casein (86). Although horses are not often used for their milk production, this large animal model might allow cross-species comparisons to better understand the mechanism of lactation.

At present, equine liver organoids have also been established, although editorial restrictions do not permit presentation of these (as yet) unpublished data. It thus remains a matter of time before an advanced *in vitro* model is available to study nutrient-related disorders in a standardized way with prognostic value for the *in vivo* situation (87).

## Discussion and future perspectives

**Figure 2:**
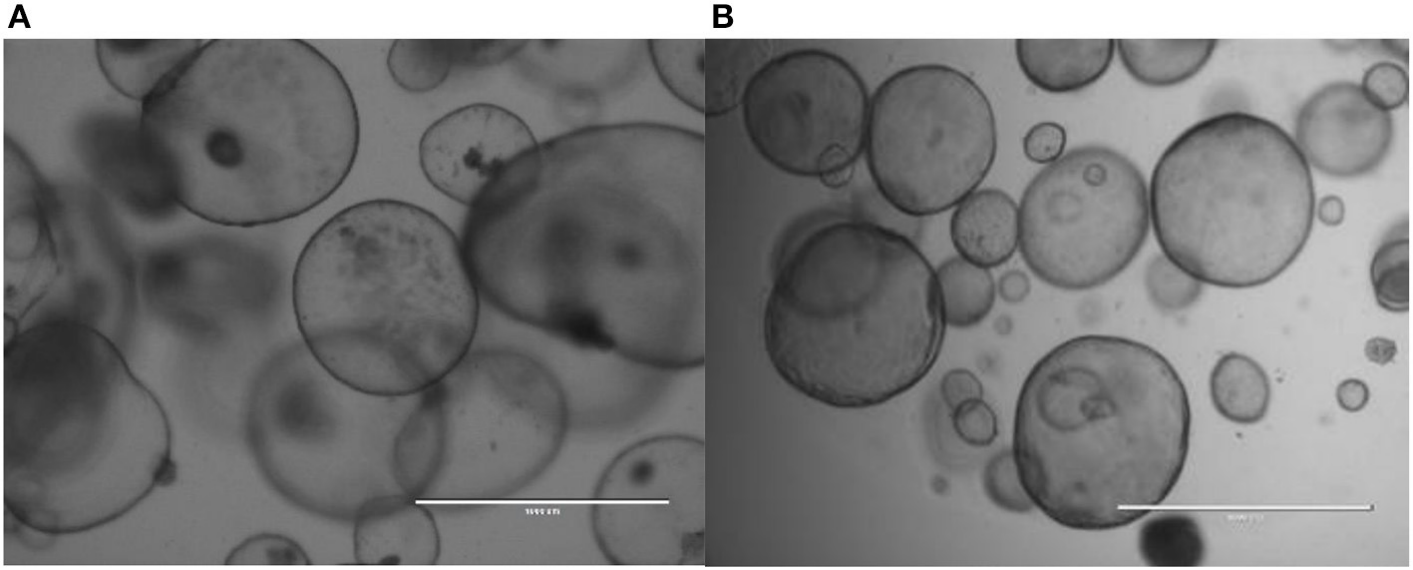
Canine pancreatic organdies **(A)** and equine liver organdies **(B)** in Matrigel. Freshly isolated cadaveric material was harvested and chopped into small pieces prior to culture. Although the initial cell cultures usually contain several cell types, the selective medium (aimed to simulate adult stem cells based on their high Wnt-signaling) after several days only stem cells survive and form these hollow structures.

Most organoid cultures (except maybe the canine skin organoids) based on adult stem cells from epithelia are cultured in the presence of stimulators for canonical Wnt-signaling (Wnt and R-spondin proteins), EGF-signaling (epidermal growth factor), and BMP (bone morphogenic proteins) in-activation (Noggin or Gremlin-1). Often, differentiation is induced by the removal of proliferative signaling molecules (Wnt-, R-spondin proteins) and addition of BMP-proteins and Notch-inhibitors. In order to start investigations into novel organoid systems, the media composition as described above (27) can be used as a starting medium, from which by step-wise depletion of individual growth factor or signaling-modifiers an optimal culture medium can be deduced. Furthermore, this study showed that for a number of veterinary relevant species recombinant human Wnt3a, R-spondin-3, and Noggin in DMEM are crucial to establish organoid cultures. This defined medium seems to be a good start to evaluate proliferation and differentiation of organoids of interest (Figures 2A, B for canine pancreatic organoids and equine liver organoids).

The potential applications of organoid technology in the field of veterinary regenerative medicine are obvious (Figure 1), but also associated with a number of ethical dilemmas (Box 1). Although disease modeling is gaining momentum, the understanding of interactions between organoids from different organs is still in its infancy in veterinary medicine. Knowledge of cellular differentiation of organoids is not yet optimal, for instance regarding hepatocyte differentiation and the use of (multi-organ) organoids in a representative drug metabolism and safety platform. This leads to a set of open questions. Are validated antibodies to detect LGR5 expression in cats and horses available? Which cell membrane markers, other than LGR5, can be used to sort single cells and measure outgrowth into organoids based on this selection marker? How large is the variation in expression of cell markers and characteristics between organoids and even within organoids? How can we guide or improve cellular differentiation, and do we need species specific growth factors to achieve this? How cost-effective is precision medicine in veterinary medicine? Once functional cell replacement of transplants can be achieved in model systems, and the COMMD1-defcient dog transplantation studies showed that this in itself is not an easy task, what is the financial burden for the owners (26)?

Box 1 **Specific ethical issues related to Matrigel and organoid ownership.**

The cell differentiation and sorting characteristics and spatially restricted lineage commitment can be addressed by a shift from 2-dimensional cultures on plastics to 3- dimensional cultures in hydrogels. These hydrogels provide structural support and possibly differentiation cues as well. The laminin-rich extracellular matrix from the Engelbreth-Holm- Swarm tumor (called Matrigel) is often used, but besides being of animal origin, and as such not FDA approved, the large batch-to-batch variation hampers experimental reproducibility (88). The search for animal-free alternatives to Matrigel is beyond the scope of this critical review, but definitely requires strong scientific input. This culture dilemma was nicely described for fetal bovine serum but is *mutatis mutandis* applicable to animal derived matrices too (89). In addition to these application limitations caused by Matrigel or animal derived alternatives, organoid technology comes with specific ethical questions, for instance related to the ownership of the cells and anonymization of patient derived material (90).

Reviews highlight the potential and advocate the use of organoid technology in veterinary medicine for personalized medicine or toxicological studies (91, 92). In today's reality disease modeling and, to a lesser degree, the study of drug metabolism are being conducted with organoid technology in the field of veterinary medicine. The few studies on transplantations, the apex of stem-cell mediated regenerative medicine, are only in the pre-clinical phase for human trials, at best. No long-term cure has been achieved but the choices of the large animal models (pigs and dogs) permit size-based extrapolation to pediatric patients. As such, the model animals are the true heroes of the one medicine concept, bridging the gap between veterinary medicine and human medicine as was recently advocated in Nature Medicine for dogs in particular (93).

## Author contributions

Both authors listed have made a substantial, direct, and intellectual contribution to the work and approved it for publication.
