# Supplementary material for: Companion animal organoid technology to advance veterinary regenerative medicine
Source: Front Vet Sci. 2023 Mar 17;10:1032835. doi: 10.3389/fvets.2023.1032835 (PMC10063859; doi:10.3389/fvets.2023.1032835)
Supplement: Supplementary file 1 [file Data_Sheet_1.docx]

**Supplementary Table 1. Reason of negative selection of hits out of the selected 91 hits.**

1. [Optogenetic control of apical constriction induces synthetic morphogenesis in mammalian tissues.](https://pubmed.ncbi.nlm.nih.gov/36104355/)

Martínez-Ara G, Taberner N, Takayama M, Sandaltzopoulou E, Villava CE, Bosch-Padrós M, Takata N, Trepat X, Eiraku M, Ebisuya M. Nat Commun. 2022 Sep 14;13(1):5400. doi: 10.1038/s41467-022-33115-0.

MDCK cells, not organoids

1. [Functionalized Nanogels with Endothelin-1 and Bradykinin Receptor Antagonist Peptides Decrease Inflammatory and Cartilage Degradation Markers of Osteoarthritis in a Horse Organoid Model of Cartilage.](https://pubmed.ncbi.nlm.nih.gov/36012214/)

Cullier A, Cassé F, Manivong S, Contentin R, Legendre F, Garcia Ac A, Sirois P, Roullin G, Banquy X, Moldovan F, Bertoni L, Audigié F, Galéra P, Demoor M. Int J Mol Sci. 2022 Aug 11;23(16):8949. doi: 10.3390/ijms23168949.

Not organoids according to Lancaster and Knoblich definition.

1. [Systemic immunosuppression promotes survival and integration of subretinally implanted human ESC-derived photoreceptor precursors in dogs.](https://pubmed.ncbi.nlm.nih.gov/35905738/)

Ripolles-Garcia A, Dolgova N, Phillips MJ, Savina S, Ludwig AL, Stuedemann SA, Nlebedum U, Wolfe JH, Garden OA, Maminishkis A, Amaral J, Bharti K, Gamm DM, Aguirre GD, Beltran WA. Stem Cell Reports. 2022 Aug 9;17(8):1824-1841. doi: 10.1016/j.stemcr.2022.06.009.

Not adult stem cells derived.

1. [Systematically quantifying morphological features reveals constraints on organoid phenotypes.](https://pubmed.ncbi.nlm.nih.gov/35705097/)

Beck LE, Lee J, Coté C, Dunagin MC, Lukonin I, Salla N, Chang MK, Hughes AJ, Mornin JD, Gartner ZJ, Liberali P, Raj A. Cell Syst. 2022 Jul 20;13(7):547-560.e3. doi: 10.1016/j.cels.2022.05.008.

MDCK cells, not organoids

1. [Bone Marrow MSC Secretome Increases Equine Articular Chondrocyte Collagen Accumulation and Their Migratory Capacities.](https://pubmed.ncbi.nlm.nih.gov/35628604/)

Contentin R, Jammes M, Bourdon B, Cassé F, Bianchi A, Audigié F, Branly T, Velot É, Galéra P. Int J Mol Sci. 2022 May 21;23(10):5795. doi: 10.3390/ijms23105795.

On Multipotent stromal cells, or Mesenchymal stem cells (MScs)

1. [Emerging enterococcus pore-forming toxins with MHC/HLA-I as receptors.](https://pubmed.ncbi.nlm.nih.gov/35259335/)

Xiong X, Tian S, Yang P, Lebreton F, Bao H, Sheng K, Yin L, Chen P, Zhang J, Qi W, Ruan J, Wu H, Chen H, Breault DT, Wu H, Earl AM, Gilmore MS, Abraham J, Dong M. Cell. 2022 Mar 31;185(7):1157-1171.e22. doi: 10.1016/j.cell.2022.02.002.

nothing to do with organoids

1. [High-Throughput Functional Analysis of CFTR and Other Apically Localized Proteins in iPSC-Derived Human Intestinal Organoids.](https://pubmed.ncbi.nlm.nih.gov/34943927/)

Xia S, Bozóky Z, Di Paola M, Laselva O, Ahmadi S, Jiang JX, Pitstick AL, Jiang C, Rotin D, Mayhew CN, Jones NL, Bear CE. Cells. 2021 Dec 4;10(12):3419. doi: 10.3390/cells10123419.

Human induced pluripotent stem cells derived organoids (iPSCs)

1. I[ntraperitoneal administration of human "Neo-Islets", 3-D organoids of mesenchymal stromal and pancreatic islet cells, normalizes blood glucose levels in streptozotocin-diabetic NOD/SCID mice: Significance for clinical trials.](https://pubmed.ncbi.nlm.nih.gov/34710142/)

Westenfelder C, Hu Z, Zhang P, Gooch A. PLoS One. 2021 Oct 28;16(10):e0259043. doi: 10.1371/journal.pone.0259043.

Not on cats, dogs or horses

1. [Multiomic analysis defines the first microRNA atlas across all small intestinal epithelial lineages and reveals novel markers of almost all major cell types.](https://pubmed.ncbi.nlm.nih.gov/34643097/)

Shanahan MT, Kanke M, Oyesola OO, Hung YH, Koch-Laskowski K, Singh AP, Peck BCE, Biraud M, Sheahan B, Cortes JE, Gong H, Sahoo DK, Cubitt R, Kurpios NA, Mochel JP, Allenspach K, McElroy SJ, Ding S, von Moltke J, Dekaney CM, Tait-Wojno ED, Sethupathy P. Am J Physiol Gastrointest Liver Physiol. 2021 Dec 1;321(6):G668-G681.

Not on cats, dogs or horses

1. [Subretinal Transplantation of Human Embryonic Stem Cell-Derived Retinal Tissue in a Feline Large Animal Model.](https://pubmed.ncbi.nlm.nih.gov/34424232/)

Occelli LM, Marinho F, Singh RK, Binette F, Nasonkin IO, Petersen-Jones SM. J Vis Exp. 2021 Aug 5;(174). doi: 10.3791/61683.

Human induced pluripotent stem cells derived organoids (iPSCs)

1. [Experimental Models for SARS-CoV-2 Infection.](https://pubmed.ncbi.nlm.nih.gov/34187969/)

Kim T, Lee JS, Ju YS. Mol Cells. 2021 Jun 30;44(6):377-383. doi: 10.14348/molcells.2021.0094.

Not on cats, dogs or horses

1. [Design, Synthesis, and Biological Evaluation of a Novel Photocaged PI3K Inhibitor toward Precise Cancer Treatment.](https://pubmed.ncbi.nlm.nih.gov/33876637/)

Zhang K, Ji M, Lin S, Peng S, Zhang Z, Zhang M, Zhang J, Zhang Y, Wu D, Tian H, Chen X, Xu H. J Med Chem. 2021 Jun 10;64(11):7331-7340. doi: 10.1021/acs.jmedchem.0c02186.

Not on cats, dogs or horses

1. [Model Systems for the Study of Malignant Melanoma.](https://pubmed.ncbi.nlm.nih.gov/33704702/)

Gregg RK. Methods Mol Biol. 2021;2265:1-21. doi: 10.1007/978-1-0716-1205-7_1.

Not on cats, dogs or horses

1. [Marine Collagen Hydrolysates Downregulate the Synthesis of Pro-Catabolic and Pro-Inflammatory Markers of Osteoarthritis and Favor Collagen Production and Metabolic Activity in Equine Articular Chondrocyte Organoids.](https://pubmed.ncbi.nlm.nih.gov/33430111/)

Bourdon B, Contentin R, Cassé F, Maspimby C, Oddoux S, Noël A, Legendre F, Gruchy N, Galéra P. Int J Mol Sci. 2021 Jan 8;22(2):580. doi: 10.3390/ijms22020580.

Not organoids according to Lancaster and Knoblich definition.

1. [Gene Regulatory Network Analysis and Engineering Directs Development and Vascularization of Multilineage Human Liver Organoids.](https://pubmed.ncbi.nlm.nih.gov/33290741/)

Velazquez JJ, LeGraw R, Moghadam F, Tan Y, Kilbourne J, Maggiore JC, Hislop J, Liu S, Cats D, Chuva de Sousa Lopes SM, Plaisier C, Cahan P, Kiani S, Ebrahimkhani MR. Cell Syst. 2021 Jan 20;12(1):41-55.e11. doi: 10.1016/j.cels.2020.11.002.

Human induced pluripotent stem cells derived organoids (iPSCs)

1. [Mammary Organoids and 3D Cell Cultures: Old Dogs with New Tricks.](https://pubmed.ncbi.nlm.nih.gov/33210256/)

Sumbal J, Budkova Z, Traustadóttir GÁ, Koledova Z. J Mammary Gland Biol Neoplasia. 2020 Dec;25(4):273-288. doi: 10.1007/s10911-020-09468-x.

Not on cats, dogs or horses

1. [Asymmetric Stratification-Induced Polarity Loss and Coordinated Individual Cell Movements Drive Directional Migration of Vertebrate Epithelium.](https://pubmed.ncbi.nlm.nih.gov/33053348/)

Lu Y, Deng R, You H, Xu Y, Antos C, Sun J, Klein OD, Lu P. Cell Rep. 2020 Oct 13;33(2):108246. doi: 10.1016/j.celrep.2020.108246.

MDCK cells, not organoids

1. [Methods to Generate Tube Micropatterns for Epithelial Morphogenetic Analyses and Tissue Engineering.](https://pubmed.ncbi.nlm.nih.gov/32939724/)

Bosch-Fortea M, Martín-Belmonte F. Methods Mol Biol. 2021;2179:227-242. doi: 10.1007/978-1-0716-0779-4_18.

MDCK cells, not organoids

1. [Animal and translational models of SARS-CoV-2 infection and COVID-19.](https://pubmed.ncbi.nlm.nih.gov/32820248/)

Johansen MD, Irving A, Montagutelli X, Tate MD, Rudloff I, Nold MF, Hansbro NG, Kim RY, Donovan C, Liu G, Faiz A, Short KR, Lyons JG, McCaughan GW, Gorrell MD, Cole A, Moreno C, Couteur D, Hesselson D, Triccas J, Neely GG, Gamble JR, Simpson SJ, Saunders BM, Oliver BG, Britton WJ, Wark PA, Nold-Petry CA, Hansbro PM. Mucosal Immunol. 2020 Nov;13(6):877-891. doi: 10.1038/s41385-020-00340-z.

Not on cats, dogs or horses

1. [Guiding Cell Network Assembly using Shape-Morphing Hydrogels.](https://pubmed.ncbi.nlm.nih.gov/32578300/)

Viola JM, Porter CM, Gupta A, Alibekova M, Prahl LS, Hughes AJ. Adv Mater. 2020 Aug;32(31):e2002195. doi: 10.1002/adma.202002195.

MDCK cells, not organoids

1. [How to orient cells in microcavities for high resolution imaging of cytokinesis and lumen formation.](https://pubmed.ncbi.nlm.nih.gov/32423649/)

Bhat A, Lu L, Wang CH, Lo Vecchio S, Maraspini R, Honigmann A, Riveline D. Methods Cell Biol. 2020;158:25-41. doi: 10.1016/bs.mcb.2020.01.002.

MDCK cells, not organoids

1. I[nfection of bat and human intestinal organoids by SARS-CoV-2.](https://pubmed.ncbi.nlm.nih.gov/32405028/)

Zhou J, Li C, Liu X, Chiu MC, Zhao X, Wang D, Wei Y, Lee A, Zhang AJ, Chu H, Cai JP, Yip CC, Chan IH, Wong KK, Tsang OT, Chan KH, Chan JF, To KK, Chen H, Yuen KY. Nat Med. 2020 Jul;26(7):1077-1083. doi: 10.1038/s41591-020-0912-6.

Not on cats, dogs or horses

1. [Spatiotemporal Changes of Cerebral Monocarboxylate Transporter 8 Expression.](https://pubmed.ncbi.nlm.nih.gov/32143555/)

Wilpert NM, Krueger M, Opitz R, Sebinger D, Paisdzior S, Mages B, Schulz A, Spranger J, Wirth EK, Stachelscheid H, Mergenthaler P, Vajkoczy P, Krude H, Kühnen P, Bechmann I, Biebermann H. Thyroid. 2020 Sep;30(9):1366-1383. doi: 10.1089/thy.2019.0544. Epub 2020 Apr 17.

MDCK cells, not organoids

1. I[s There a Trojan Horse to Aggressive Pancreatic Cancer Biology? A Review of the Trypsin-PAR2 Axis to Proliferation, Early Invasion, and Metastasis.](https://pubmed.ncbi.nlm.nih.gov/32064449/)

Søreide K, Roalsø M, Aunan JR. J Pancreat Cancer. 2020 Feb 6;6(1):12-20. doi: 10.1089/pancan.2019.0014.

Not on cats, dogs or horses

1. [The YrbE phospholipid transporter of Salmonella enterica serovar Typhi regulates the expression of flagellin and influences motility, adhesion and induction of epithelial inflammatory responses.](https://pubmed.ncbi.nlm.nih.gov/31829769/)

Verma S, Prescott RA, Ingano L, Nickerson KP, Hill E, Faherty CS, Fasano A, Senger S, Cherayil BJ. Gut Microbes. 2020 May 3;11(3):526-538. doi: 10.1080/19490976.2019.1697593.

Not on cats, dogs or horses

1. [Cerebral Organoid Models for Neurotropic Viruses.](https://pubmed.ncbi.nlm.nih.gov/31580050/)

Antonucci J, Gehrke L. ACS Infect Dis. 2019 Dec 13;5(12):1976-1979. doi: 10.1021/acsinfecdis.9b00339.

Not on cats, dogs or horses

1. C[haracterization of Adult Canine Kidney Epithelial Stem Cells That Give Rise to Dome-Forming Tubular Cells.](https://pubmed.ncbi.nlm.nih.gov/31495275/)

Chen TC, Neupane M, Chien SJ, Chuang FR, Crawford RB, Kaminski NE, Chang CC. Stem Cells Dev. 2019 Nov 1;28(21):1424-1433. doi: 10.1089/scd.2019.0049.

MDCK cells, not organoids

1. [Production, Characterization, and Function of Pseudoislets from Perinatal Canine Pancreas.](https://pubmed.ncbi.nlm.nih.gov/31450972/)

Czernichow P, Reynaud K, Kerr-Conte J, Furthner E, Ravassard P. Cell Transplant. 2019 Dec;28(12):1641-1651. doi: 10.1177/0963689719869004.

isolated islets not organoids

1. [A Versatile Biosynthetic Hydrogel Platform for Engineering of Tissue Analogues.](https://pubmed.ncbi.nlm.nih.gov/31402634/)

Klotz BJ, Oosterhoff LA, Utomo L, Lim KS, Vallmajo-Martin Q, Clevers H, Woodfield TBF, Rosenberg AJWP, Malda J, Ehrbar M, Spee B, Gawlitta D. Adv Healthc Mater. 2019 Oct;8(19):e1900979. doi: 10.1002/adhm.201900979.

Not on cats, dogs or horses

1. [Organs by design: can bioprinting meet self-organization?](https://pubmed.ncbi.nlm.nih.gov/31348016/)

Martin I, Malda J, Rivron NC. Curr Opin Organ Transplant. 2019 Oct;24(5):562-567. doi: 10.1097/MOT.0000000000000679.

Not on cats, dogs or horses

1. [Evaluation of mouse enteroids as a model for Lawsonia intracellularis infection.](https://pubmed.ncbi.nlm.nih.gov/31324204/)

Resende TP, Medida RL, Guo Y, Vannucci FA, Saqui-Salces M, Gebhart C. Vet Res. 2019 Jul 19;50(1):57. doi: 10.1186/s13567-019-0672-9.

Not on cats, dogs or horses

1. [Transplantation of Human Embryonic Stem Cell-Derived Retinal Tissue in the Subretinal Space of the Cat Eye.](https://pubmed.ncbi.nlm.nih.gov/31210100/)

Singh RK, Occelli LM, Binette F, Petersen-Jones SM, Nasonkin IO. Stem Cells Dev. 2019 Sep 1;28(17):1151-1166. doi: 10.1089/scd.2019.0090.

human organoids

1. [Tropism of influenza B viruses in human respiratory tract explants and airway organoids.](https://pubmed.ncbi.nlm.nih.gov/31097520/)

Bui CHT, Chan RWY, Ng MMT, Cheung MC, Ng KC, Chan MPK, Chan LLY, Fong JHM, Nicholls JM, Peiris JSM, Chan MCW. Eur Respir J. 2019 Aug 15;54(2):1900008. doi: 10.1183/13993003.00008-2019.

Not on cats, dogs or horses

1. [Transfer of Functional Cargo in Exomeres.](https://pubmed.ncbi.nlm.nih.gov/30956133/)

Zhang Q, Higginbotham JN, Jeppesen DK, Yang YP, Li W, McKinley ET, Graves-Deal R, Ping J, Britain CM, Dorsett KA, Hartman CL, Ford DA, Allen RM, Vickers KC, Liu Q, Franklin JL, Bellis SL, Coffey RJ. Cell Rep. 2019 Apr 16;27(3):940-954.e6. doi: 10.1016/j.celrep.2019.01.009.

MDCK cells, not organoids

1. [Epithelial polarization in 3D matrix requires DDR1 signaling to regulate actomyosin contractility.](https://pubmed.ncbi.nlm.nih.gov/30760555/)

Søgaard PP, Ito N, Sato N, Fujita Y, Matter K, Itoh Y. Life Sci Alliance. 2019 Feb 13;2(1):e201800276. doi: 10.26508/lsa.201800276.

MDCK cells, not organoids

1. [p63-Dependent Dickkopf3 Expression Promotes Esophageal Cancer Cell Proliferation via CKAP4.](https://pubmed.ncbi.nlm.nih.gov/30181180/)

Kajiwara C, Fumoto K, Kimura H, Nojima S, Asano K, Odagiri K, Yamasaki M, Hikita H, Takehara T, Doki Y, Morii E, Kikuchi A. Cancer Res. 2018 Nov 1;78(21):6107-6120. doi: 10.1158/0008-5472.CAN-18-1749.

MDCK cells, not organoids

1. [Nitazoxanide Inhibits Human Norovirus Replication and Synergizes with Ribavirin by Activation of Cellular Antiviral Response.](https://pubmed.ncbi.nlm.nih.gov/30104275/)

Dang W, Xu L, Ma B, Chen S, Yin Y, Chang KO, Peppelenbosch MP, Pan Q. Antimicrob Agents Chemother. 2018 Oct 24;62(11):e00707-18. doi: 10.1128/AAC.00707-18

Not on cats, dogs or horses

1. [Mutant p53-Expressing Cells Undergo Necroptosis via Cell Competition with the Neighboring Normal Epithelial Cells.](https://pubmed.ncbi.nlm.nih.gov/29949757/)

Watanabe H, Ishibashi K, Mano H, Kitamoto S, Sato N, Hoshiba K, Kato M, Matsuzawa F, Takeuchi Y, Shirai T, Ishikawa S, Morioka Y, Imagawa T, Sakaguchi K, Yonezawa S, Kon S, Fujita Y. Cell Rep. 2018 Jun 26;23(13):3721-3729. doi: 10.1016/j.celrep.2018.05.081.

Not on cats, dogs or horses

1. [Aberrant endocytosis leads to the loss of normal mitotic spindle orientation during epithelial glandular morphogenesis.](https://pubmed.ncbi.nlm.nih.gov/29903910/)

Clancy JW, Sheehan CS, Tricarico CJ, D'Souza-Schorey C. J Biol Chem. 2018 Aug 3;293(31):12095-12104. doi: 10.1074/jbc.RA117.001640.

MDCK cells, not organoids

1. [Dia1-dependent adhesions are required by epithelial tissues to initiate invasion.](https://pubmed.ncbi.nlm.nih.gov/29437785/)

Fessenden TB, Beckham Y, Perez-Neut M, Ramirez-San Juan G, Chourasia AH, Macleod KF, Oakes PW, Gardel ML. J Cell Biol. 2018 Apr 2;217(4):1485-1502. doi: 10.1083/jcb.201703145.

MDCK cells, not organoids

1. [Intestinal organoids containing poly(lactic-co-glycolic acid) nanoparticles for the treatment of inflammatory bowel diseases.](https://pubmed.ncbi.nlm.nih.gov/29226615/)

Davoudi Z, Peroutka-Bigus N, Bellaire B, Wannemuehler M, Barrett TA, Narasimhan B, Wang Q. J Biomed Mater Res A. 2018 Apr;106(4):876-886. doi: 10.1002/jbm.a.36305.

Not on cats, dogs or horses

1. [Whole-Mount Immunocytochemistry in Xenopus.](https://pubmed.ncbi.nlm.nih.gov/29192091/)

Klymkowsky MW. Cold Spring Harb Protoc. 2018 Jan 2;2018(1). doi: 10.1101/pdb.prot097295.

Not on cats, dogs or horses

1. C[onsequences of congenital Zika virus infection.](https://pubmed.ncbi.nlm.nih.gov/29080429/)

Platt DJ, Miner JJ. Curr Opin Virol. 2017 Dec;27:1-7. doi: 10.1016/j.coviro.2017.09.005.

Not on cats, dogs or horses

[44. Adaptive self-organization in the embryo: its importance to adult anatomy and to tissue engineering.](https://pubmed.ncbi.nlm.nih.gov/29023694/)

Davies JA. J Anat. 2018 Apr;232(4):524-533. doi: 10.1111/joa.12691.

Not on cats, dogs or horses

1. [Ninein is essential for apico-basal microtubule formation and CLIP-170 facilitates its redeployment to non-centrosomal microtubule organizing centres.](https://pubmed.ncbi.nlm.nih.gov/28179500/)

Goldspink DA, Rookyard C, Tyrrell BJ, Gadsby J, Perkins J, Lund EK, Galjart N, Thomas P, Wileman T, Mogensen MM. Open Biol. 2017 Feb;7(2):160274. doi: 10.1098/rsob.160274.

MDCK cells, not organoids

1. S[ystem-Wide Modulation of HECT E3 Ligases with Selective Ubiquitin Variant Probes.](https://pubmed.ncbi.nlm.nih.gov/26949039/)

Zhang W, Wu KP, Sartori MA, Kamadurai HB, Ordureau A, Jiang C, Mercredi PY, Murchie R, Hu J, Persaud A, Mukherjee M, Li N, Doye A, Walker JR, Sheng Y, Hao Z, Li Y, Brown KR, Lemichez E, Chen J, Tong Y, Harper JW, Moffat J, Rotin D, Schulman BA, Sidhu SS. Mol Cell. 2016 Apr 7;62(1):121-36. doi: 10.1016/j.molcel.2016.02.005.

Not on cats, dogs or horses

1. [Comparative lipid analysis in the normal and cancerous organoids of MDCK cells.](https://pubmed.ncbi.nlm.nih.gov/26783265/)

Yoshizaki H, Ogiso H, Okazaki T, Kiyokawa E. J Biochem. 2016 Jun;159(6):573-84. doi: 10.1093/jb/mvw001.

MDCK cells, not organoids

1. [Regulation of Ripply1 expression in MDCK organoids.](https://pubmed.ncbi.nlm.nih.gov/26514726/)

Yoshizaki H, Kuwajima Y, Minato H, Kiyokawa E. Biochem Biophys Res Commun. 2015 Dec 4-11;468(1-2):337-42. doi: 10.1016/j.bbrc.2015.10.099.

MDCK cells, not organoids

1. R[NA Interference Screen to Identify Kinases That Suppress Rescue of ΔF508-CFTR.](https://pubmed.ncbi.nlm.nih.gov/25825526/)

Trzcińska-Daneluti AM, Chen A, Nguyen L, Murchie R, Jiang C, Moffat J, Pelletier L, Rotin D. Mol Cell Proteomics. 2015 Jun;14(6):1569-83. doi: 10.1074/mcp.M114.046375.

MDCK cells, not organoids

[49. A combination of Wnt and growth factor signaling induces Arl4c expression to form epithelial tubular structures.](https://pubmed.ncbi.nlm.nih.gov/24562386/)

Matsumoto S, Fujii S, Sato A, Ibuka S, Kagawa Y, Ishii M, Kikuchi A. EMBO J. 2014 Apr 1;33(7):702-18. doi: 10.1002/embj.201386942.

MDCK cells, and mouse organoids

1. [Chimaerin suppresses Rac1 activation at the apical membrane to maintain the cyst structure.](https://pubmed.ncbi.nlm.nih.gov/23284959/)

Yagi S, Matsuda M, Kiyokawa E. PLoS One. 2012;7(12):e52258. doi: 10.1371/journal.pone.0052258.

MDCK cells, not organoids

1. C[omputational investigation of epithelial cell dynamic phenotype in vitro.](https://pubmed.ncbi.nlm.nih.gov/19476639/)

Kim SH, Park S, Mostov K, Debnath J, Hunt CA. Theor Biol Med Model. 2009 May 28;6:8. doi: 10.1186/1742-4682-6-8.

MDCK cells, not organoids

1. [The c-Src tyrosine kinase associates with the catalytic domain of ErbB-2: implications for ErbB-2 mediated signaling and transformation.](https://pubmed.ncbi.nlm.nih.gov/16170374/)

Kim H, Chan R, Dankort DL, Zuo D, Najoukas M, Park M, Muller WJ. Oncogene. 2005 Nov 17;24(51):7599-607. doi: 10.1038/sj.onc.1208898.

MDCK cells, not organoids

1. [Calcifying odontogenic cyst associated with complex odontoma: case report and review of the literature.](https://pubmed.ncbi.nlm.nih.gov/15876968/)

Gallana-Alvarez S, Mayorga-Jimenez F, Torres-Gómez FJ, Avellá-Vecino FJ, Salazar-Fernandez C. Med Oral Patol Oral Cir Bucal. 2005 May-Jul;10(3):243-7.

Not on cats, dogs or horses
